# P-2034. Evaluation of Implementing Text-Messaging Based Medication Reminders to Improve Adherence to Antiretrovirals in People Living with HIV

**DOI:** 10.1093/ofid/ofaf695.2198

**Published:** 2026-01-11

**Authors:** Laura Mitten, Athina Schmidt, Diana F Clarke, Melanie Berry, Michael Maiullari, Alejandra Salazar, Mary R Bartkus, Archana Asundi

**Affiliations:** Boston Medical Center, Boston, MA; Boston Medical Center, Boston, MA; Boston Medical Center, Boston, MA; GILEAD, Boston, Massachusetts; Boston Medical Center, Boston, MA; Boston Medical Center, Boston, MA; Boston Medical Center, Boston, MA; Boston Medical Center, Boston, MA

## Abstract

**Background:**

Maintaining virologic suppression through antiretroviral treatment (ART) is essential for optimizing the health outcomes of people living with HIV (PWH) and reducing transmission risk. Virologic suppression requires strict adherence to ART regimens. Many PWH find oral regimen adherence to be challenging; however, text reminders are a potential tool that can support adherence. To determine if implementation of text reminders from healthcare providers supported adherence, viral suppression, and patient satisfaction, we implemented a customizable SMS-based medication text reminder service offered to individuals receiving HIV care in order determine impact.

**Figure:**
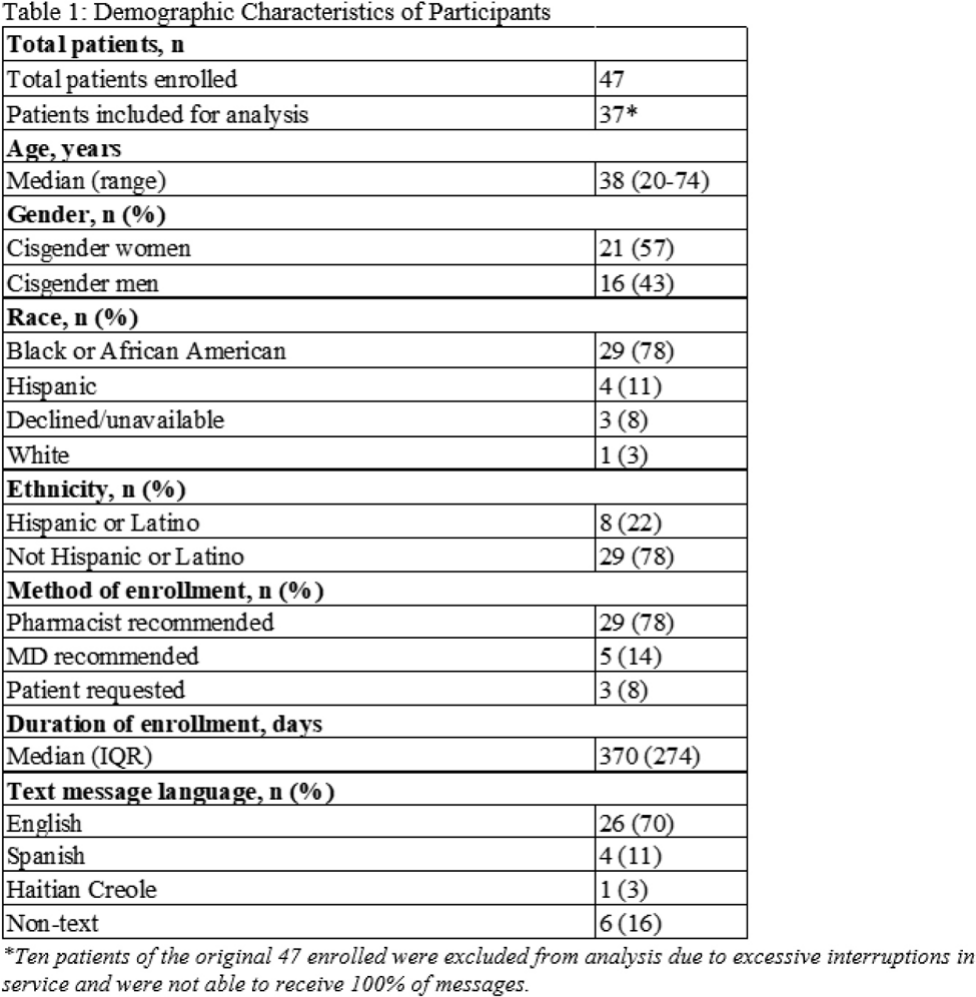

**Figure:**
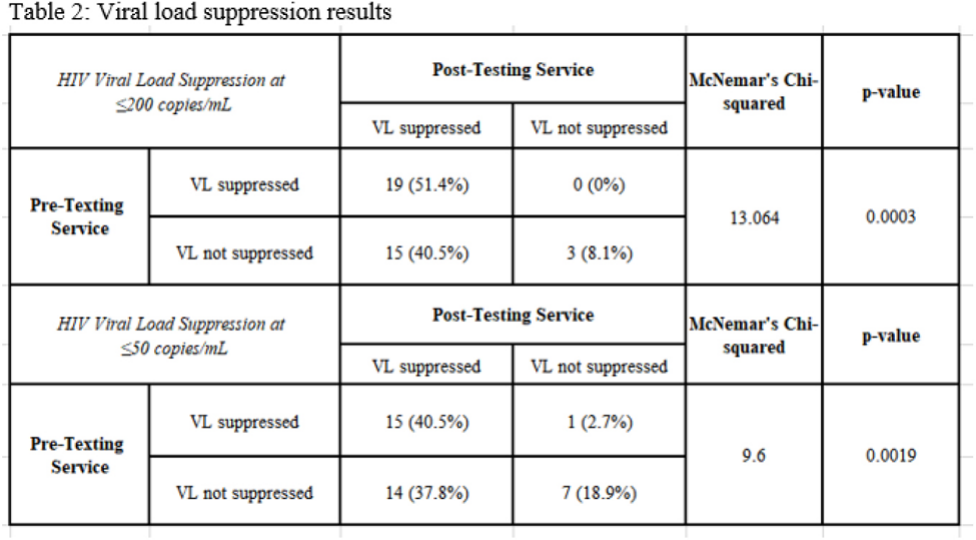

**Methods:**

Patients (≥18 years, PWH) at Boston Medical Center were enrolled in the texting service by clinical staff from 02/19/22 to 03/08/24 (N=47). Participants could fully customize the timing, content, and language of their reminders. Demographic and viral load (VL) data were extracted from the medical record, including baseline VL before enrollment and most recent VL as of 09/30/24 or closest to service disenrollment. McNemar’s test was used to assess association between VL suppression and exposure to the texting service (N=37). Virologic suppression was defined as < 200 copies/mL. Perceived adherence and satisfaction were assessed through optional text surveys among participants enrolled ≥30 days (N=21); four also completed follow-up phone interviews.

**Figure:**
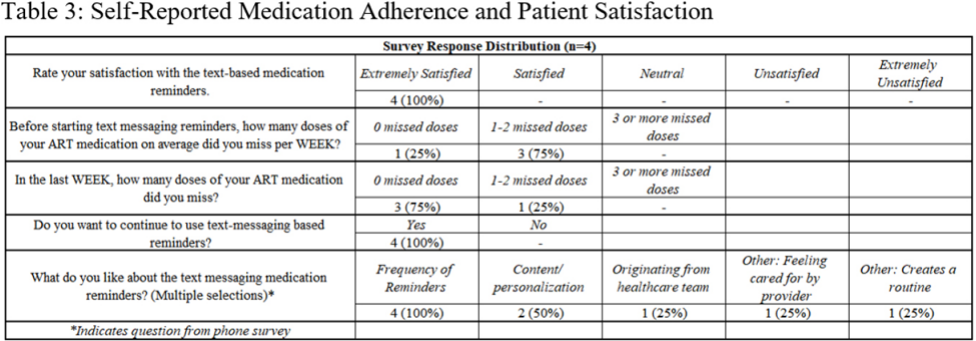

**Results:**

Table 1 shows the N=37 individuals enrolled in the text message service with baseline demographics. Median baseline VL was 188 copies/mL with 49% (N=18) unsuppressed at baseline (Table 2). We found a statistically significant difference between pre- and post-intervention VL suppression for patients who enrolled in the reminder service (p=0.0003). Results were also significant among the same patients using a 50 copies/mL threshold (p=0.0019). Survey respondents self-reported improved or maintained medication adherence levels and high satisfaction (Table 3).

**Conclusion:**

Results suggest the implementation of customizable care team-delivered text medication reminders can be used as a tool to achieve virologic suppression, ART adherence, and continued patient satisfaction.

**Disclosures:**

Melanie Berry, PharmD, Gilead: Smpliyee Michael Maiullari, PharmD, BCIDP, Gilead Sciences: Advisor/Consultant|ViiV Healthcare: Advisor/Consultant Archana Asundi, MD, DayZero Diagnostics: Grant/Research Support|Gilead Sciences: Advisor/Consultant|Gilead Sciences: Grant/Research Support|Theratechnologies: Grant/Research Support|Viiv Healthcare: Grant/Research Support

